# Trained immunity induced by in vivo peptide-based STAT6 inhibition prevents ragweed allergy in mice

**DOI:** 10.1186/s13223-021-00542-5

**Published:** 2021-04-21

**Authors:** Husheem Michael, Yuanyi Li, Yufa Wang, Christine T. McCusker

**Affiliations:** Meakins-Christie Laboratories, McGill University and the McGill University Health Care-Research Institute, Block E, RI-MUHC, EM3.2219, 1001 Boulevard Décarie, Montréal, QC H4A 3J1 Canada

**Keywords:** Ragweed, Trained immunity, STAT6 mucosal vaccination, Allergic airways disease, Asthma, Murine model

## Abstract

**Background:**

Trained immunity is the ability of the innate immune system to form immune memory responses to provide support the formation of appropriate adaptive responses. Allergic airways disease (AAD) is a maladapted immune response to allergens, initiated and maintained by the type 2 (T2) inflammatory pathway. It is predicated by the elaboration of cytokines IL-4 and IL-13 and follows activation of the STAT6 transcription factor.

**Objective:**

To investigate the role of trained immunity in mucosal immune responses following neonatal vaccination with the STAT6 inhibitory peptide (STAT6-IP), in preventing the development of ragweed-induced AAD.

**Methods:**

We demonstrate that transfer of CD4^+^ T cells or dendritic cells (DC) from STAT6-IP vaccinated wild-type BALB/c mice to naïve mice, that were subsequently chronically exposed to sensitizing doses of ragweed allergen, is sufficient to prevent development of T2 responses in recipients.

**Results:**

Our results demonstrate significant reductions in; airways hyperresponsiveness (AHR); ragweed-specific IgE; pulmonary inflammation; T2 cytokines; and inflammatory gene expressions in recipient mice. Expression of IDO, TGFβ and T regulatory cells were all significantly increased. Anti-TGFβ treatment during the ragweed sensitization phase re-constituted the pro-inflammatory T2 immune response. We show that tolerance can be attained via DC trained in the STAT6-IP-mediated tolerant milieu. This effect is not restricted to a particular allergen and does not require antigen-mediated T cell activation prior to transfer.

**Conclusion:**

Adoptive transfer experiments suggest that STAT6-IP treatment trains dendritic and cells to mediate tolerant immunity to chronic ragweed exposure in the airways. This indicates that early transient STAT6-inhibition constitutes an effective immunomodulatory airways allergy preventative strategy.

**Supplementary Information:**

The online version contains supplementary material available at 10.1186/s13223-021-00542-5.

## Introduction

Early environmental stimuli immunomodulates mucosal responses resulting in changes to lifetime risk of the development of allergic airways disease (AAD) [1–3]. Prevention strategies of allergen avoidance have been generally futile [4–7] however environmental modulation aimed at promoting regulatory immune pathways in the airways have shown promise [3, 8].

Fetal and neonatal immune responses differ significantly than in children and adults. Both the innate and adaptive immune systems are quite impaired in neonates. In neonates, there are increased numbers of naïve T and NK cells with decreased expression of some proinflammatory cytokines [8]. During childhood, induction of the allergic phenotype, involving the differentiation of allergen-specific T cells into the inflammatory type 2 (T2) phenotype, may result from a failure to properly polarize T cell subsets. [9, 10] Preferential activation of T2 cells upon novel allergen exposure follows the local expression of IL-4 during allergen presentation n by dendritic cells (DCs) [10–13]. During neonatal period, immature DCs originate in the bone marrow, migrate to the airways and mature under the influence of the local lung environment [11]. Mucosal exposures to complex antigenic (microbial) environments lead to the maturation of tolerogenic DCs while reduced or absent microbial exposures may result in the T2 promoting milieu [11, 14, 15].

IL-4 and IL-13, canonical T2 cytokines, bind to receptors that exhibit a common IL-4Rα subunit and binding of these cytokines to their cognate receptor results in recruitment and tyrosine phosphorylation of the STAT6 transcription factor [16]. We previously reported the development of a chimeric inhibitory peptide, STAT6-IP, which abrogates allergen-induced airways hyperresponsiveness (AHR) and inflammation in established acute ovalbumin and chronic ragweed experimental models of allergic airways disease (AAD) [17, 18]. In subsequent work, we postulated that inhibition of T2-mediated cell activation, during a critical development window in neonates, would promote programming of immune tolerance and prevent allergy development. We showed that when neonatal mice were inoculated with STAT6-IP intranasally, they developed tolerance to subsequent allergen exposure, with either ovalbumin or ragweed allergens. This tolerant phenotype was associated with low allergen-specific IgE, reduced T2 cytokine expression, and the significant increase in the LAP + Treg + cells subset and expression of TGFβ. Moreover, OVA-sensitized CD4^+^ T cells or CD11c^+^ DCs taken from STAT6-IP vaccinated OVA-transgenic (DO11.10) mice, when transferred into naïve mice, transferred this regulatory phenotype following OVA stimulation. This regulatory phenotype was TGFβ-dependent [19].

Trained immunity is a recently evolving concept that defines the ability of the immune system to form innate immune memory. Numerous studies have shown that trained immunity plays a critical role in inflammatory and neurodegenerative diseases in humans [20]. Moreover, group 2 innate lymphoid cells have also been suggested to exhibit aspects of trained immunity responses following stimulation with *Aspergillus oryzae* protease and papain allergens [21]. Machiels et al. [22] have also demonstrated that the severity of house dust mite-induced asthma was decreased in the lungs of mice that had previously been chronically infected with gammaherpesvirus and that resident regulatory innate cells are responsible for mediating this anti-inflammatory effect.

In the present study we harvested and transferred CD4^+^ T cells or DC from wild-type BALB/c mice inoculated with STAT6-IP as neonates. Recipient naïve BALB/c mice maintained immunotolerance to allergen even when chronically exposed to sensitizing doses of ragweed. Moreover, ‘tolerogenic’ T cells or DCs showed increased expression of IDO and proliferation of ragweed sensitized T2 cells was inhibited by the ‘tolerogenic’ CD4^+^ T cells in vitro. These data demonstrated that early mucosal STAT6-IP vaccination trained DCs and promoted Treg formation capable of influencing subsequent immune responses through the creation of active airways tolerance to the allergen. Moreover, in the present study we have established the generalizability of our previous findings. We have demonstrated that ragweed-sensitized CD4^+^ T cells or CD11c^+^ DCs harvested from STAT6-IP vaccinated Balb/c mice, when transferred into naïve Balb/c mice, had transferred regulatory phenotype following ragweed stimulation. In contrast to our previous work, T cells from these animals were not enriched for antigen-specificity. We show that transfer of tolerance is achievable following prolonged sensitization. These data suggest that STAT6-IP treatment induces dendritic cell-mediated trained immunity leading to tolerance when exposed subsequently to allergen in the airways.

## Material and methods

### Peptide synthesis

STAT-6-inhibitory peptides (STAT6-IP or IP) and STAT6-control peptides (STAT6-CP or CP) were synthesized by the University of Calgary Integrated Peptide Services (Calgary Alberta, Canada). Peptides are comprised of a derivative of the TAT protein transduction domain, YARAAARQARA [23]. This sequence is coupled to an 8 amino acid sequence surrounding tyrosine 641 in murine STAT6. In the STAT6-IP the tyrosine residue is phosphorylated (GRG*YVSTT) and in the control STAT6-CP peptide, this residue is replaced by a phenylalanine (GRGFVSTT). Peptides were amidated at the carboxyl terminus and purified by RP-HPLC and analyzed by MS. Peptide sequences, uptake, stability in vivo and inhibitory effects of the STAT6-IP have been detailed previously in an acute OVA-induced [17] and chronic ragweed induced models of AAD [18].

### Animals

Four to six weeks old BALB/c mice were obtained from Harlan-Spraque Dawley (Indianapolis, IN). Mice were bred and housed in a conventional animal facility at the Meakins-Christie Laboratories. For each experimental condition a minimum of 6–8 animals was used. All studies followed the Canadian Council of Animal Care (CCAC) guidelines and were approved by the Animal Care Committee of McGill University, animal protocol number 4658.

### Peptide vaccination

Beginning on the third day of life, 50 µg of either STAT6-IP or STAT6-CP, or PBS, was administered intranasally (IN) in a volume of 10µL daily for 5 days [19].

### Allergen sensitization and challenge

For chronic ragweed-induced AAD: Awake 4–6 week old animals were sensitized 5 days per week for 5 weeks by local instillation of 10 μl in each nare of 1% (10 mg/ml) ragweed (Giant Ragweed, extracted from *Ambrosia trifida*, Sigma-Aldrich, St Louis MO) in PBS as previously described [18]. After a 2 week rest, mice were challenged with 1% (10 mg/ml, dry weight) Amba I (Short Ragweed, extracted from *ambrosia artemisiifolia* pollen grains, Greer, Lenoir NC) in PBS once per day for 5 consecutive days and assayed 24 h following the final challenge.

### Assessment of airway hyperresponsiveness (AHR) by methacholine challenge

Total respiratory resistance was measured 24 h after ragweed challenge using a small animal ventilator (FlexiVent, SCIREQ, Montreal, Qc Canada) as described in detail elsewhere [17, 18]. Briefly, mice were deeply anesthetized with xylazine hydrochloride (NovaPharm, Toronto, On, Canada) followed by sodium pentobarbital (Sandoz, Montreal Qc Canada, CEVA, Santé Animale, France) and paralyzed with pancuronium bromide. Heart rate was monitored by EKG to ensure adequate anesthesia throughout the procedure. Mice were ventilated quasi-sinusoidally with a tidal volume of 8 ml/kg and a respiratory rate of 150 breaths/min. Methacholine was given by nebulizer (Aeroneb, SCIREQ, Montreal, Qc) at doses of 3.125, 25, and 50 mg/ml and maximal resistance were obtained for each graded dose of methacholine.

### Histological analysis and differential cell count in bronchoalveolar lavage fluid (BAL)

Pulmonary histopathology was performed as described previously [24]. Briefly, lungs were slowly inflated with 1 ml formalin, isolated, and then placed entirely in formalin. The specimens were embedded in paraffin, and 0.5 μm sections were cut. Representative images are shown at 100 × magnification. Immediately post-mortem, lungs were lavage twice in situ with 0.75 ml of ice-cold saline. The returned fractions were pooled and centrifuged. The pellets were used for total and differential cell counts as previously described [24]. Briefly, red blood cells were lysed using ACK lysing buffer (PharM Lyse™, BD Biosciences, Mississauga, ON). Cells were spun onto glass slides and stained using the Diffquick method (Dade Behring Inc IL). Differential cell counts were obtained manually under light microscopy. 40 × power fields were counted per slide and means obtained.

### Measurement of ragweed-specific IgE in serum

Blood was obtained from mice post-challenge by post-mortem intracardiac puncture. Ragweed-specific IgE was assayed as described previously [17, 18, 25]. Specific IgE was quantified by ELISA (Pharmingen OptEIA™ IgE Kit, San Diego CA) with the following modifications: serum was incubated overnight at 4 ºC with protein G sepharose beads (Amersham Bioscience, Piscataway, NJ) before loading onto ELISA plates that had been previously coated with short ragweed. Standards were plated as per OptEIA protocol. Samples were incubated overnight at 4 ºC and ELISA performed as per protocol. The specific antibodies were quantified with mouse IgE ELISA sets (BD Biosciences Pharmingen, San Diego, CA).

### Splenocyte culture and cytokine assessment

Single-cell suspensions were prepared from the whole spleen as described previously [18, 19]. Briefly, erythrocytes were lysed by ACK lysing buffer and the washed splenocytes were resuspended at 5 × 10^6^ cells/ml in complete RPMI-1640 medium with 10% heat-inactivated FBS, 2 mM l-glutamine, 50 µM 2-ME, 100 U/ml penicillin, 100 µg/ml streptomycin sulfate. Splenocytes were then cultured for 4 days at 37 °C with 5% CO_2_ in the presence of Amba I (10 mg/ml). Supernatants were stored at − 80 ºC before quantifying cytokine levels by ELISA. Murine IL-4, IL-10, IL-13, TGFβ, and IFNγ levels in supernatants of the ragweed-stimulated cultured splenocytes were quantified using ELISA kits purchased from eBioscience Inc (San Diego, CA) as per manufacturer’s instructions.

### T cell suppression assay

CD4^+^ T cells were purified from spleens of ragweed sensitized mice and incubated with carboxyfluorescein succinimidyl ester (CFSE; 5 μM) for 8 min at room temperature [19]. Cells were then washed and resuspended in media for 15 min. CFSE-labeled CD4^+^ T cells (responder T cells) were mixed with CD4^+^ T cells, isolated as described above, from STAT6-IP treated or sham control mice in ratio of 1:1 to 1:8 and cocultured in 24-well plates. Cells were stimulated with 10 mg/mL ragweed in the presence of 2 × 10^5^ purified CD11c^+^ DCs from allergen naïve mice. After 3 days, cells were harvested, and CFSE dilution was analyzed by means of flow cytometry [19].

### Adoptive transfer of CD4^+^ T cells or CD11c^+^ DCs

For adoptive transfer experiments, 5 × 10^6^ MACS purified CD4^+^ T cells, from IP- or CP-vaccinated or PBS sham vaccinated BALB/c mice, were injected into the tail vein of naïve BALB/c recipients in a volume of 50 µL of normal saline [19]. Twenty-four hours later recipient mice were sensitized and challenged with ragweed as per protocol. In other experiments 5 × 10^5^ MACS purified CD11c^+^ DCs from these same donor mice were injected intratracheally into naïve BALB/c recipients in a volume of 50 µL of normal saline [19]. Twenty-four hours later these mice were sensitized and challenged with ragweed as per protocol [19].

### In vivo treatment with the anti-TGFβ antibody

Animals were treated with 50 µg anti-TGFβ pan-neutralizing antibody or isotype control IN every second day during sensitization with the allergen as previously described [19]. Monoclonal pan-anti-TGFβ1,-β2,-β3 (MAB 1835) and normal mouse IgG1 isotype control (MAB002) were purchased from R&D System (Minneapolis, MN).

### RNA extraction and real-time RT-PCR

To assess mRNA levels of inflammatory genes in lung tissues, the total cellular RNA was extracted from the lungs using RNeasy Mini Kit, according to the manufacturer’s protocol (Qiagen Inc, Toronto, On, Canada), and reverse transcribed using SuperScript II reverse transcriptase (Invitrogen Life Technologies, Carlsbad, CA). Real-time quantitative PCR was performed with a LightCycler System (Roche Applied Sciences, Indianapolis IN) using QuantiTect SYBR Green (Qiagen Inc, Toronto, On). Each target was quantified using four tenfold serial dilutions of standards prepared from PCR amplicons that had been gel purified and quantified using a Fluorochem 8000 imaging system and AlphaEase software (Alpha Innotech, San Jose, CA). Values were then normalized to GAPDH that had been reverse transcribed, PCR amplified, and quantified. The following mouse primers were used [19]: Muc-2, sense 5′-GCT GAC GAG TGG TTG GTG AAT G-3′ and antisense 5′-GAT GAG GTG GCA GAC AGG AGA C-3′, Muc-5ac, sense 5′-CAG CCG AGA GGA GGG TTT GAT CT-3′ and antisense 5′-AGT CTC TCT CCG CTC CTC TCA AT-3′. Eotaxin-1 (CCL11), sense 5′-GGG CAG TAA CTT CCA TCT GTC TCC-3′ and antisense 5′-CAC TTC TTC TTG GGG TCA GC-3′. GAPDH, sense 5′-GCC ATG GAC TGT GGT CAT GA-3′ and antisense 5′-TTC ACC ACC ATG GAG AAG GC-3′. IDO, sense 5′-GTA CAT CAC CAT GGC GTA TG-3′ and antisense 5′-CGA GGA AGA AGC CCT TGT C-3'.

### Flow cytometric analysis

Surface markers and FoxP3 were evaluated by flow cytometry [19]. Splenocytes cultured for 4 days with Amba I were stimulated for 5 h with murine anti-CD3 (0.5 μg/ml; clone 2C11, BD Pharmingen, Mississauga, On, Canada) and monensin (GolgiStop; BD Biosciences, Mississauga, On, Canada), according to the manufacturer’s instructions. Cells were then washed, permeabilized with saponin (Perm/Wash; BD Biosciences) and fixed in formaldehyde and PBS (Cytofix/Cytoperm; BD Biosciences) for 30 min. Cell surface markers were stained with labeled rat anti-mouse CD4, CD25, TGFβ, or Foxp3 on ice for 30 min and then washed with PBS and analyzed on a FACSCalibur (BD, San Jose, CA). Ten thousand cells were counted from each sample and data were analyzed with BD flow cytometer and Cell Quest Software. Cells were also stained with respective isotype control antibodies.

### Statistical analysis

The results represented ± SEM of six to eight replicates per experiment and each experiment was repeated at least three times. The data was analyzed using one-way analysis of variance followed by Tukey’s post hoc tests for individual group comparisons. A value of *p* < 0.05 was considered significant.

## Results

### T2 immune responses are inhibited by transfer of CD4^+^ T cells from STAT6-IP inoculated mice.

**Fig. 1:**
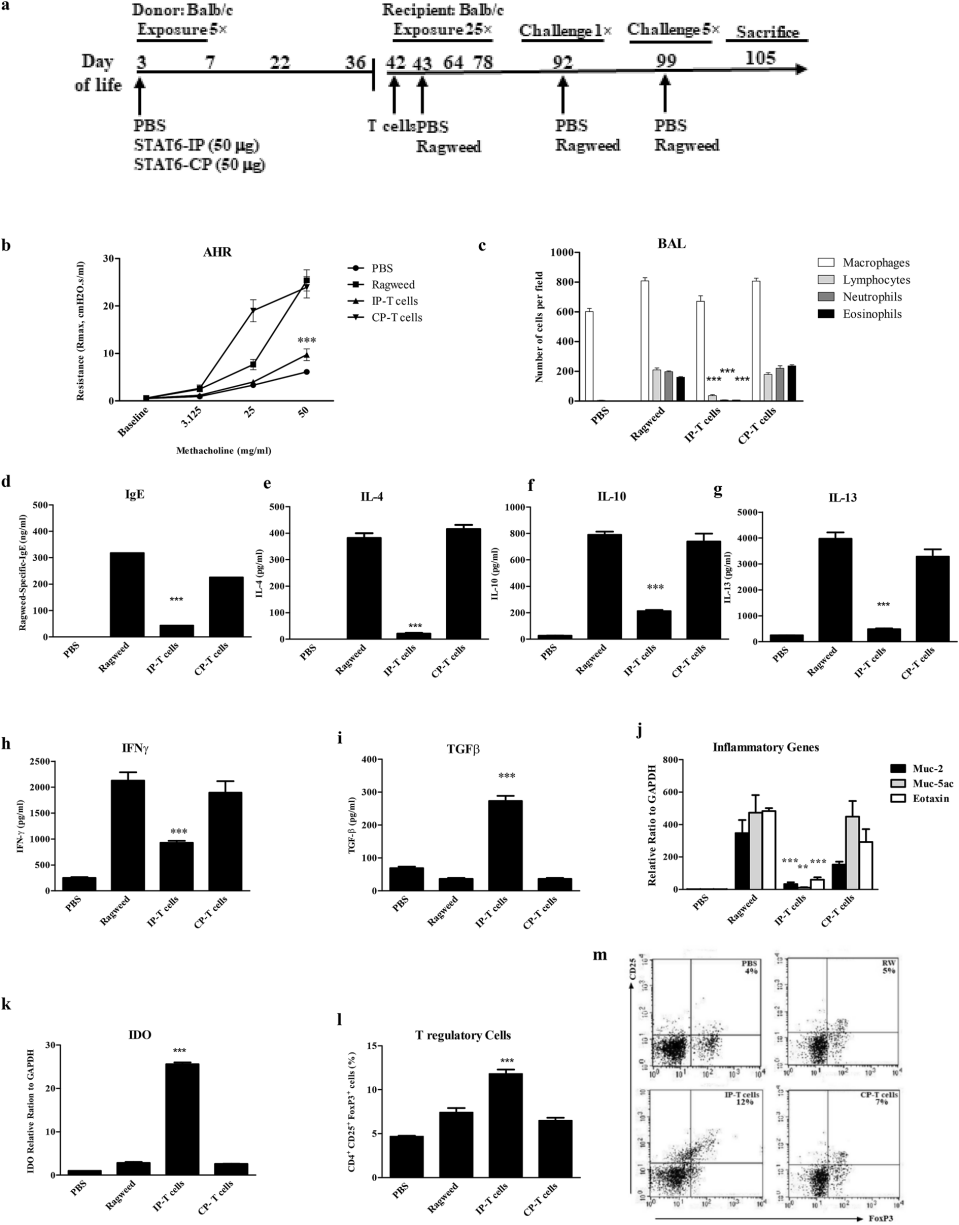
Adoptively transferred STAT6-IP CD4^+^T cells prevented allergic airways disease (AAD) in naïve recipient mice. BALB/c mice were vaccinated with STAT6-IP or STAT6-CP as described in methods. **a** CD4^+^ T cells were then isolated from STAT6-IP (IP-T cells) or STAT6-CP (CP-T cells) vaccinated mice and injected into the tail vein of naïve BALB/c mice. Twenty-four hours later recipient mice were sensitized and challenged with ragweed as per protocol. **b** Bronchial airway hyperresponsiveness (AHR) to methacholine following challenge with ragweed was assessed in recipient mice using the Flexivent small animal ventilator. PBS (filled circle), Ragweed (Filled square), IP-T cells (filled upward triangle), and CP-T cells (Filled downward triangle), respectively. **c** Differential cell counts from recipient mice were obtained from recovered bronchial alveolar lavage (BAL) fluid and stained with Diff-Quick method. White column denotes macrophages (open square), lymphocytes (filled square) light grey, neutrophils (filled square) dark grey, and eosinophils (filled square) black. **d** Serum OVA-specific-IgE from recipient animals determined by ELISA. Splenocytes from STAT6-IP, -CP or sham vaccinated mice recipient mice were cultured in triplicates in the presence of ragweed for four days and **e** IL-4, **f** IL-10, **g** IL-13, **h** IFNγ and **i** TGFβ were quantified from supernatants by ELISA. **j** Inflammatory genes (Muc-2, Muc-5ac, Eotaxin-1) were investigated in whole lung homogenates from mice using RT-PCR. **k** IDO expression was assessed in lung cells of vaccinated mice by RT-PCR. **l**, **m,** Frequency of T regulatory (CD4^+^CD25^+^FOXP3^+^) cells was investigated in splenocytes from vaccinated mice determined by flow cytometry analysis. Each experiment included 6–8 animals per group and experiment replicated at least three times. Error bars indicated the standard error of mean. IP group compared with ragweed group, **p < 0.01 ***p < 0.001 were considered significant

STAT6-IP (IP) or a control peptide (CP) were instilled daily in the nares of awake 3–8 day old mice (Fig. 1a). The STAT6-IP differs from the STAT6-CP via a single substitution of the phosphotyrosine for a phenylalanine [17]. CD4^+^ T cells from neonatal STAT-6-IP (IP-T cells) exposed mice were then harvested from spleen and transferred into naïve mice to determine if the observed AAD protective effect was transferrable by these cells. T2 immune responses were then assessed following chronic sensitization (5 weeks) and challenge with ragweed allergen (Fig. 1b–m). IP-T cell recipient immune response was compared with CP-T cell recipient (CP-T), allergic (ragweed) mice and naïve (PBS) mice. AHR to methacholine were significantly increased in both the CP-T and ragweed groups while IP-T responded similarly to the PBS non-allergic mice (Fig. 1b). IP-T recipient mice also demonstrated minimal inflammatory cell influx assessed in the BAL (Fig. 1c) however, BAL samples showed lower expressions of cytokines (data not shown). There was minimal production of serum ragweed-specific IgE in IP-T recipient mice as compared with CP-T and ragweed groups (Fig. 1d). Cytokine expression from splenocyte cultures derived from IP-T cell recipient mice showed significantly reduced levels of IL-4, IL-10, IL-13, and IFNγ following stimulation with ragweed whereas TGFβ level was significantly elevated (Fig. 1e–i). Moreover, the frequency of CD4^+^TGFβ^+^ cells was increased in IP-T mice (Additional file 1: Figure S1). Expressions of T2-associated inflammatory genes from whole lung homogenates, MUC2, MUC5ac and Eotaxin were significantly reduced in IP-T group as compared with CP-T and ragweed groups (Fig. 1j). IDO expression was significantly elevated in pulmonary cells derived from IP-T group (Fig. 1k). The frequency of splenocyte CD4^+^CD25^+^Foxp3^+^ T cells was also increased in IP-T recipient mice compared with other treatment groups (Fig. 1l, m). T cells from vaccinated mice inhibited proliferation of T cells from ragweed allergic mice. In contrast proliferation of these cells was robust in the presence of CP-T cells. Taken together these data suggest that neonatal STAT6-IP vaccination promotes the formation of tolerogenic CD4^+^ T cells which retain this anti-T2-inflammatory activity when adoptively transferred to naïve mice.

### T2 immune responses are inhibited by transfer of CD11c^+^ DC from STAT6-IP inoculated mice.

**Fig. 2:**
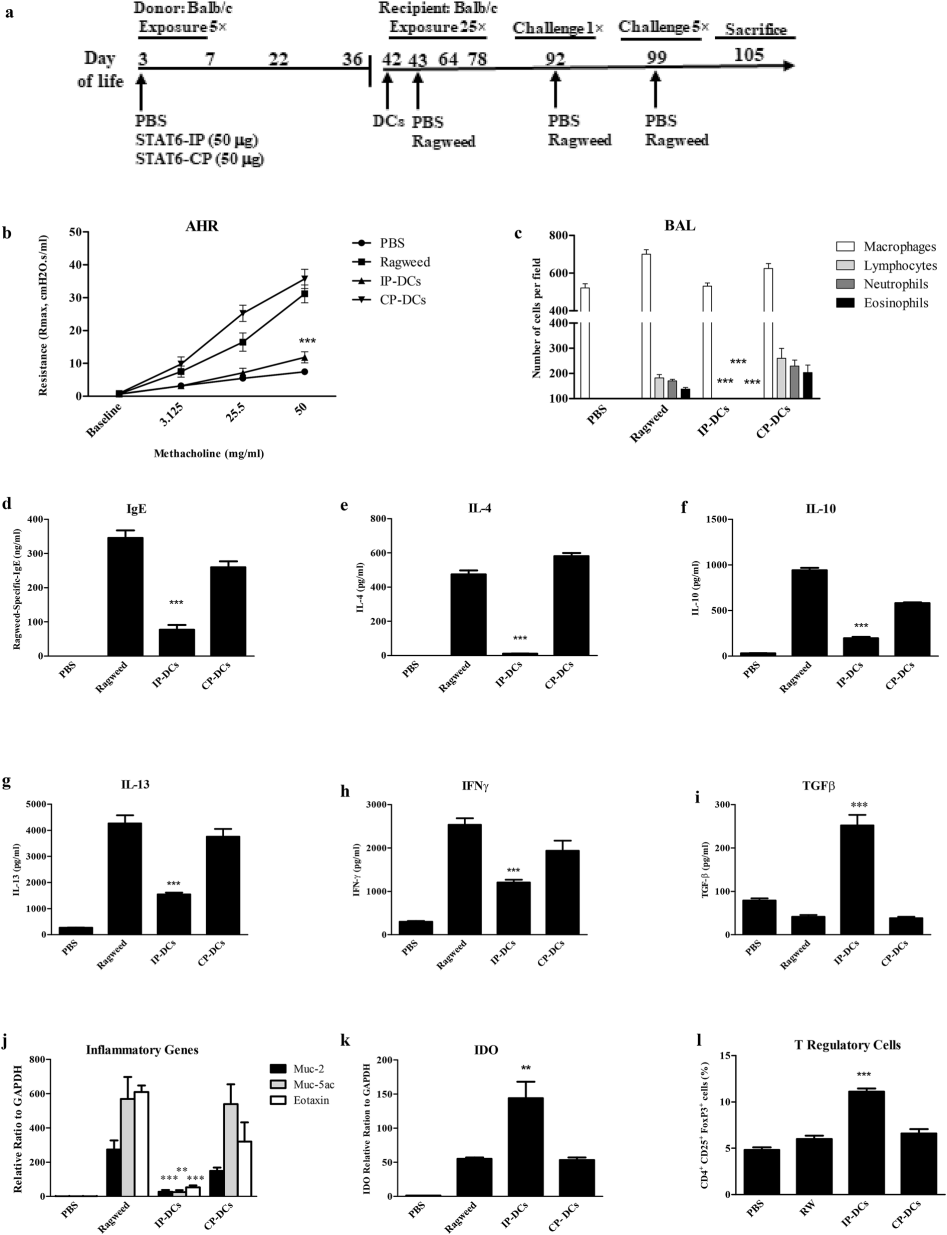
Adoptively transferred STAT6-IP CD11c^+^ DCs prevented allergic airways disease (AAD) in naïve recipient mice. BALB/c mice were vaccinated with STAT6-IP or STAT6-CP as described in methods. **a** CD11c^+^ DCs were then isolated from STAT6-IP (IP-DCs cells) or STAT6-CP (CP-DCs) vaccinated mice and adoptively transferred intratracheally into naïve BALB/c mice. Twenty-four hours later recipient mice were sensitized and challenged with ragweed as per protocol. **b** Bronchial airway hyperresponsiveness (AHR) to methacholine following challenge with ragweed was assessed in recipient mice using the Flexivent small animal ventilator. PBS (filled circle), Ragweed (filled square), IP-T cells (filled upward triangle), and CP-T cells (filled downward triangle), respectively. **c** Differential cell counts from recipient mice were obtained from recovered bronchial alveolar lavage (BAL) fluid and stained with Diff-Quick method. White column denotes macrophages (open square), lymphocytes (filled square) light grey, neutrophils (filled square) dark grey, and eosinophils (filled square) black. **d** Serum OVA-specific-IgE from recipient animals determined by ELISA. Splenocytes from STAT6-IP, -CP or sham vaccinated mice recipient mice were cultured in triplicates in the presence of ragweed for four days and **e** IL-4, **f** IL-10, **g** IL-13, **h** IFNγ and **i** TGFβ were quantified from supernatants by ELISA. **j** Inflammatory genes (Muc-2, Muc-5ac, Eotaxin-1) were investigated in whole lung homogenates from mice using RT-PCR. **k** IDO expression was assessed by RT-PCR in lung cells of vaccinated mice. **l** The frequency of T regulatory (CD4^+^CD25^+^FOXP3^+^) cells was investigated in splenocytes from vaccinated mice determined by flow cytometry analysis. Each experiment included 6–8 animals per group and experiment replicated at least three times. Error bars indicated the standard error of mean. IP group compared with ragweed group, **p < 0.01, and ***p < 0.001 were considered significant

**Fig. 3:**
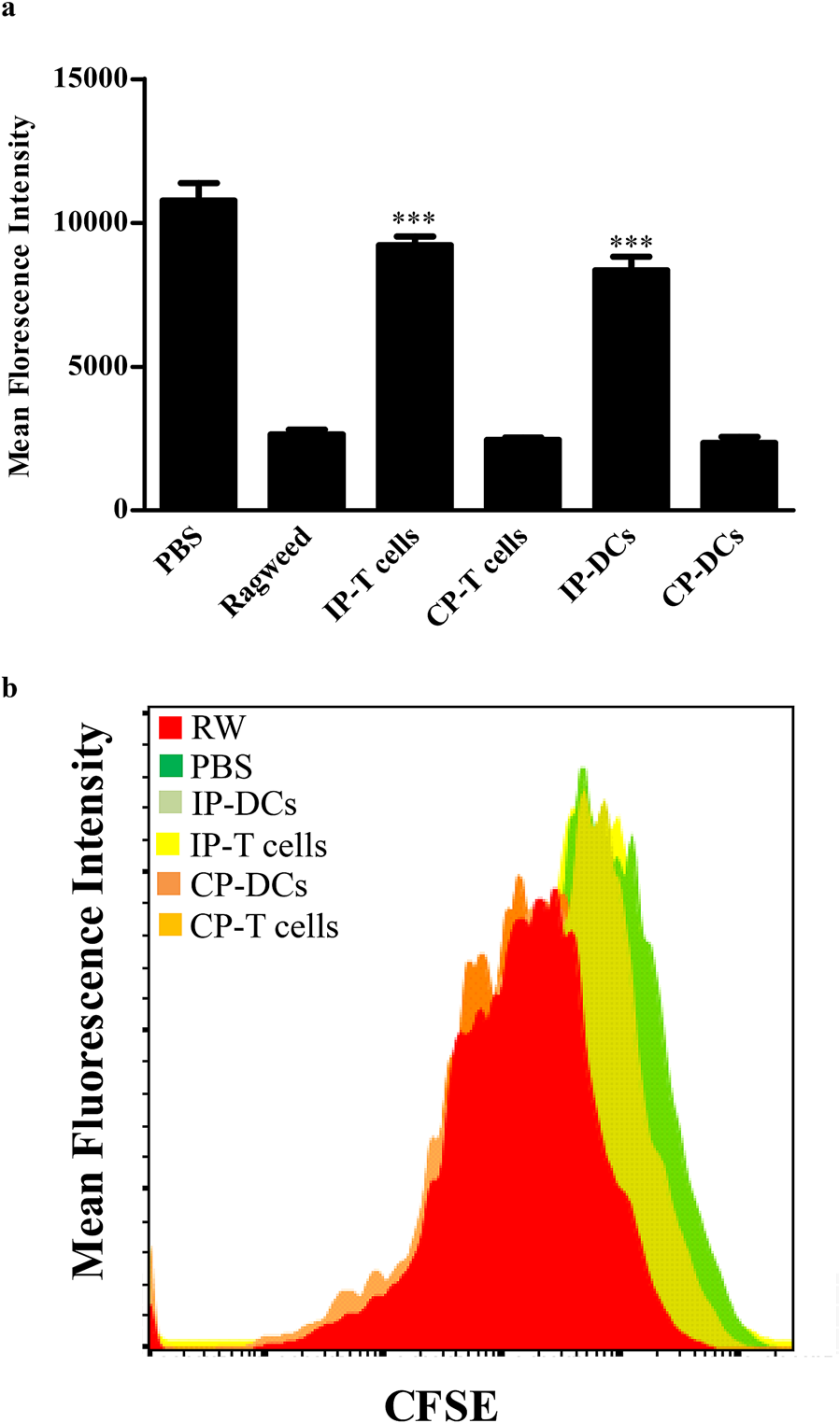
CD4^+^ T cells from IP vaccinated DCs or T cells mice suppressed the proliferation of ragweed sensitized T cells. CSFC stained CD4^+^ T cells, purified from spleens of ragweed sensitized mice, were mixed with CD4^+^ T cells, isolated from IP-T, IP-DC treated or sham control mice and cocultured in 24-well plates. Cells were stimulated with 10 mg/mL ragweed in the presence of 2 × 10^5^ purified CD11c^+^ DCs from allergen naïve mice. After 3 days, cells were harvested, and CFSE dilution was analyzed by means of flow cytometry. PBS control responder cells were derived from non-sensitized mice. Cells from IP-T and IP-DC recipient animals inhibited ragweed-mediated proliferation of T cells. Each experiment included 6–8 animals per group and experiment replicated at least three times. Error bars indicated the standard error of mean. IP group compared with ragweed group, ***p < 0.001 were considered significant

**Fig. 4:**
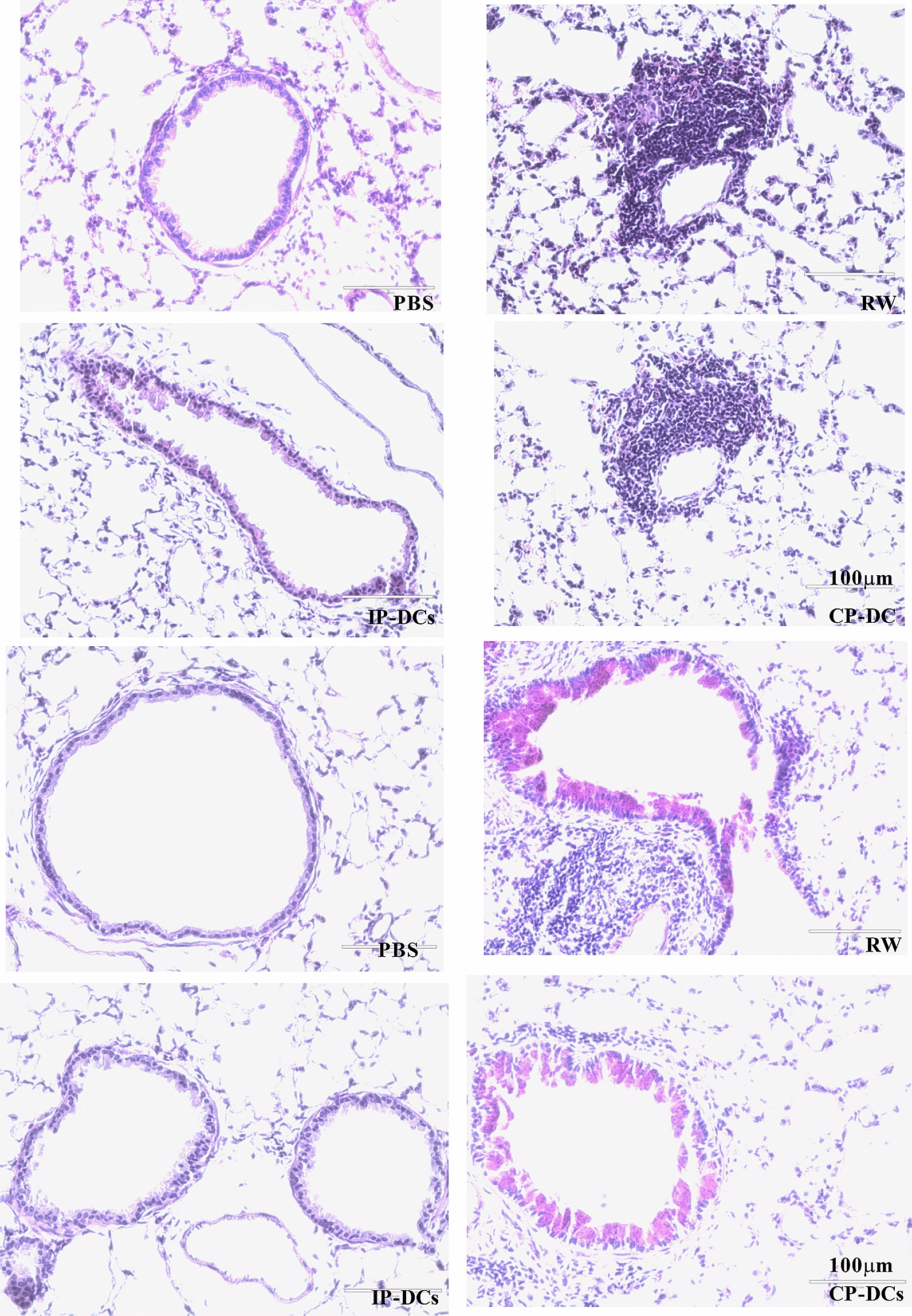
STAT6-IP vaccinated DCs cells mice reduced the inflammatory cells in lung tissues. Lung sections were stained with H&E and Periodic Acid Schiff stain. Inflammatory cells and mucus-positive cells were identified with × 100 magnification. Each experiment included 6–8 animals per group and experiment replicated at least three times

We next determined if CD11c^+^ dendritic cells (DCs) from neonatal STAT-6-IP exposed mice could mediate the AAD protective effect in naïve mice. CD11c^+^ DCs from IP- or CP- exposed BALB/c mice were isolated and adoptively transferred intratracheally into naïve animals that were then ragweed sensitized and challenged and allergic responses were evaluated (Fig. 2a). Similar to IP-T recipients, IP-DCs recipient mice showed airway responses to methacholine were comparable with naïve mice while both CP-DCs and ragweed groups had significant AHR (Fig. 2b). IP-DC recipients, when compared to CP-DCs recipient or allergic mice, had minimal inflammatory cell influx in BAL (Fig. 2c) and low levels of ragweed-specific IgE in serum (Fig. 2d). Splenocyte cultures from IP-DC recipient mice also produced significantly lower levels of IL-4, IL-10, IL-13, and IFNγ following stimulation with ragweed whereas the TGFβ levels were significantly elevated (Fig. 2e-i). Moreover, the frequency of CD4^+^TGFβ^+^ cells was increased in IP-DC mice (Additional file 1: Figure S1). Expression of T2 inflammatory genes were significantly reduced in IP-DC group as compared with CP-DCs or ragweed groups (Fig. 2j). IDO expression, often associated with tolerant DC phenotype, was significantly elevated in IP-DC recipients (Fig. 2k) [26, 27]. The frequency of CD4^+^CD25^+^Foxp3^+^ T cells was also increased in IP-DC mice (Fig. 1l). As shown in Fig. 3, CD4^+^ T cells from IP-DC mice inhibited proliferation of ragweed sensitized T cells in vitro. We further investigated if IP treatment ameliorated pulmonary pathology, for this purpose lungs sections were stained with H&E and periodic acid Schiff. As shown in Fig. 4, there were significantly accumulation of inflammatory cells in lung while with IP treatment these inflammatory cells were reduced compared with CP or RW treated mice. IP treated mice were comparable with PBS treated mice. Taken together these data suggest that neonatal STAT6-IP vaccination promotes the formation of tolerant DCs which also retained the capacity to promote, de novo, active T cell-mediated immune tolerance to allergen when adoptively transferred to naïve mice.

### Induction of STAT6-IP-mediated neonatal tolerance is TGFβ-dependent

**Fig. 5:**
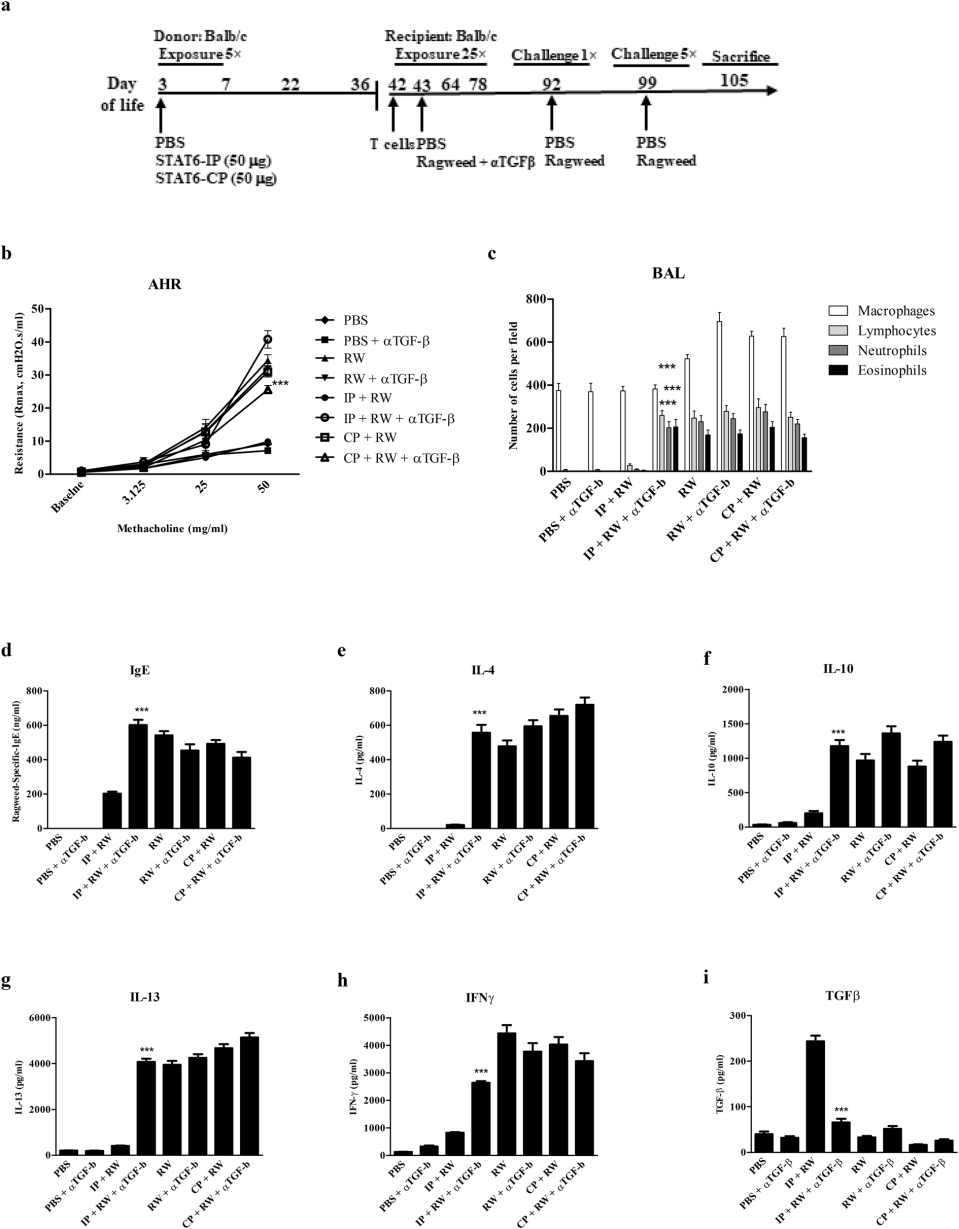
Trained immunity mediated bySTAT6-IP vaccination is TGFβ dependent. Naïve BALB/c mice received CD4^+^ T cells from STAT6-IP or CP vaccinated donors. **a** Recipients mice were sensitized with ragweed as described in the methods. Pan anti-TGFβ antibody (αTGFβ) was given intranasally every other day during sensitization in some recipient animals and T2 responses were assessed post-challenge. **b** Bronchial airway hyperresponsiveness (AHR) to methacholine following challenge with ragweed (RW) was assessed using the Flexivent small animal ventilator. PBS (filled diamond), PBS + αTGFβ (filled square), RW (filled upward triangle), RW + αTGFβ (filled downward triangle), IP + RW (filled circle), IP + RW + αTGFβ (open circle), CP + RW (open square), and CP + RW + αTGFβ (open upward triangle). **c** Differential cell counts were obtained from recovered BAL fluid and stained with Diff-Quick method. White column denotes macrophages (open square), lymphocytes (filled square) light grey, neutrophils (filled square) dark grey, and eosinophils (filled square) black. **d** Serum OVA-specific-IgE from recipient animals determined by ELISA. Splenocytes from STAT6-IP, -CP or sham vaccinated mice recipient mice were cultured in triplicates in the presence of ragweed for four days and **e** IL-4, **f** IL-10, **g** IL-13, **h** IFNγ and **i** TGFβ were quantified from supernatants by ELISA. For all experiments 6–8 animals per group and experiments were replicated at least three times. Error bars indicated the standard error of mean. IP + αTGFβ group compared with IP groups, ***p < 0.001

In allergen-induced responses from STAT6-IP vaccinated mice TGFβ levels were significantly elevated relative to those from naive (PBS), allergic (ragweed), or CP-vaccinated mice. To determine if TGFβ was required for the observed tolerance to allergen in IP-T recipient mice, a pan neutralizing anti-TGFβ antibody was given to these animals during sensitization with ragweed (Fig. 5a). Anti-TGFβ treatment nullified the protective effects vaccination when delivered at the time of ragweed sensitization. AHR (Fig. 5b), BAL fluid inflammatory cell influx (Fig. 5c), and ragweed-specific IgE (Fig. 5d) were all elevated in IP-vaccinated mice when TGFβ was neutralized during ragweed sensitization. Also, cytokine levels from splenocyte cultures showed that neutralization of TGFβ in IP-T mice at the time of ragweed sensitization restored IL-4, IL-13, IL-10, and IFNγ while, as expected, TGFβ levels were reduced to baseline (Fig. 5e–i). We assumed that expressions of T2-associated inflammatory genes, MUC2, MUC5ac and Eotaxin and IDO would be similar as described in Fig. 1j. Neutralizing TGFβ in CP-vaccinated or sham vaccinated ragweed sensitized mice had no effect on ragweed induced airway inflammatory responses. While the activity of TGFβ is critically important for the induction of tolerance, once established the role of TGFβ in perpetuating the phenotype appears to be minimal.

## Discussion

Developmental immune programming is effective in the neonatal period. Several studies have shown that late fetal and early postnatal immune responses are fundamentally different from adults and interventions during the critical window could train DCs and T cell responses with long-term mucosal and systemic influences [14, 28–30]. Moreover, postnatal stimuli in the airways can lead to the migration of immature bone marrow DCs to the airways [10]. Polarization of these stimulated DCs are affected by the local environment and the type of existing stimuli [9–11, 31].

In this study we have shown persistent modulation of trained mucosal immune responses can be achieved through adoptive transfer. STAT6-IP vaccination resulted in the polarization of CD4^+^ T cells with an increase in functional Treg cells, and CD11c^+^ tolerant DCs and when either CD4^+^ T cells or CD11c^+^ DCs were transferred to naïve hosts, the tolerant phenotypes were recapitulated in the recipients. Tolerance to aeroallergen was manifested as a reduced allergen-induced specific IgE, either reduced or absent airways inflammation, T1 and T2 cytokine elaboration, and reduced AHR (Figs. 1, 2). Interestingly, IL-10 cytokines expression was reduced in our investigations. These outcomes were comparable to naïve non-allergen exposed mice. IP-T and -DC recipient mice also had increased TGFβ levels (Figs. 1h, 2h, Additional file 1: Figure S1). We have demonstrated that following transfer of STAT6-IP treated DC and T cells there are decreases in T2 cytokines upon ragweed stimulation. We have also shown decreased levels of both IL-10 and IFN-gamma (Fig. 2). While in neutrophilic asthma, evidence supports a role for resident IL-10 producing macrophages [32] the models presented herein examine eosinophilic asthma. Increased in both IL-10 and TGFβ were found increased in both lung and in cultured splenocytes in this model following STAT6-IP therapy.

When TGFβ activity was neutralized in IP-T recipient mice, at the time of ragweed sensitization, the protective effects of transfer, on development of AAD, were eliminated (Fig. 3). These data suggest that there is an overall net decrease in inflammatory responses to ragweed stimulation in treated animals possibly via the observed increase in Treg cells in these mice.

We have previously shown that intranasal application of STAT6-IP in neonatal and adult mice resulted in a reduction in allergen-induced AHR and inflammation in allergic animals [17–19]. In the present study, neonates were vaccinated with STAT6-IP and 4 weeks later cells were harvested for transfer. The positive transfer of the tolerant response suggests the development of stable immunity which is non-allergen specific. Inhibition of STAT6 appears to polarize DCs and ultimately influence T cells, before allergen-mediated activation supporting the hypothesis that a form of trained immunity leads to tolerance in this model.

The concept of trained innate immunity has been proposed following the observation that certain live vaccines such as smallpox and BCG protected vaccines from other infections through “enhanced” immunity [33]. Evidence also suggests that Toll-like receptor (TLR) stimulation with ligands such as flagellan will influence DC-mediated responses to other microbes [34, 35]. We have shown that mucosal LPS stimulation induced TH1 and tolerant immune responses in our model of ADD [25]. In the present study we show that inhibition of T2 immunity early in life promotes the stable formation of tolerogenic DCs and CD4^+^ T cell pathways.

Transfer of tolerant phenotypes-induced airway immune responses to ragweed challenge were characterized by increased expression of IDO (Figs. 1j, 2j). IDO is an inducible enzyme that catalyzes the rate-limiting first step in tryptophan catabolism along the kynurenine pathway [36]. Tryptophan deprivation by IDO consumption inhibits T-cell activation while kynurenine derivatives and O_2_-free radicals, regulate T-cell proliferation and survival [26, 36]. Also, IDO expressing DCs have been shown to inhibit T cell proliferation [37].

Tolerogenic DCs have been shown to modulate T cell responses by inducing T cell anergy, T cell apoptosis, and induction of Tregs [30]. Moreover, they may induce the development of T cell tolerance through a variety of mechanisms including production of cytokines such as IL-10 and TGFβ as well as inhibition of T cell proliferation and cytokine expression and via the production of IDO [30]. We hypothesize that STAT6-IP prevents the activation of the T2 pathways through DC-T cell interaction. Our data suggests that the absence of T2 signaling in this immunological synapse leads to increased TGFβ production. We are currently investigating the mechanisms involved. In the present study we have shown active tolerance induction to aeroallergens can be achieved by adoptive transfer of STAT6-IP DCs tolerant phenotype associated with increased allergen-induced TGFβ production. Numerous studies have shown that TGFβ suppresses the activity, proliferation and/or survival of various inflammatory cells including T2 cells, B cells, macrophages, and eosinophils [38–42]. Our data demonstrated that induction of aeroallergen transference of tolerance by the STAT6-IP DCs was TGFβ-dependent (Fig. 3) and associated with amelioration in the Foxp3^+^ T cell population (Figs. 1k, 2k).

Moreover, transfer of the tolerant phenotype was achieved by CD4 + T or CD11c + cell adoptive transfer into naïve recipients and T cells from STAT6-IP vaccinated mice actively suppressed proliferation of T cells from ragweed sensitized mice (Fig. 3). These T cell data suggest that the T cells generated following STAT6-IP vaccination were functional Tregs. Also, the additional data showing the effectiveness of DCs adoptive transfer as a means to prevent allergic sensitization suggests that STAT6-IP vaccination also resulted in the stable polarization or training of DCs which promoted functional allergen tolerance (Fig. 2) [43]. There was higher yield of splenocytes as compared with lymph nodes in mice, hence splenocytes were used for these studies. It is interesting to note that while only in the nares of the mice were exposed to STAT6-IP, splenocytes derived DC to transfer the protective non-allergic phenotype to naïve mice. This suggests that DC-mediated trained immunity not only provides local protection from subsequent disease but also may modulate systemic immune responses. In these complex in vivo experimental systems, it is possible that other cell types, including airway macrophages and structural cells contributed to the development of this trained immunity [35]. Future studies will address how STAT6- dependent inhibition mechanism contributes to reduce AAD in neonatal mice. This study confirms the potential of STAT6-IP therapy in AAD, demonstrating that the effects are not restricted to a particular allergen. These data, together with those of the adoptive transfer experiments, presented herein, suggest that STAT6-IP treatment induces dendritic and T cell-mediated trained immunity leading to tolerance when exposed subsequently to allergen in the airways.

In conclusion, this study has shown that mucosal inhibition of the STAT6 pathway trains DC and T cell responses to aeroallergen. The immune response stimulated in naïve IP-T or IP-DC mice, following allergen exposure in the airways leads ultimately to active tolerance and prevention of allergic airways disease.

## Supplementary Information

**Additional file 1: Figure S1.** The frequency of CD4+TGFβ+ cells was increased in IP-T or IP-DC recipient mice. CD4+ T cells or DC were isolated from STAT6-IP or STAT6-CP vaccinated mice as described in the methods and adoptive transferred to naïve recipients. CD4+ T cells were then isolated from STAT6-IP (IP-DCs/IP-T cells) or STAT6-CP (CP-DCs/CP-T cells) recipients following ragweed exposure and the frequency of cells was determined by flow cytometry. Each experiment included 6–8 animals per group and experiment replicated at least three times. Error bars indicated the standard error of mean. IP group compared with ragweed group, ***p < 0.001 were considered significant.

## Data Availability

The data that support the findings of this study are available from the corresponding author upon reasonable request.
