# Supplementary material for: Trained immunity induced by in vivo peptide-based STAT6 inhibition prevents ragweed allergy in mice
Source: Allergy Asthma Clin Immunol. 2021 Apr 21;17:42. doi: 10.1186/s13223-021-00542-5 (PMC8059037; doi:10.1186/s13223-021-00542-5)
Supplement: Supplementary file 1 — Additional file 1: Figure S1. The frequency of CD4+TGFβ+ cells was increased in IP-T or IP-DC recipient mice. CD4+ T cells or DC were isolated from STAT6-IP or STAT6-CP vaccinated mice as described in the methods and adoptive transferred to naïve recipients. CD4+ T cells were then isolated from STAT6-IP (IP-DCs/IP-T cells) or STAT6-CP (CP-DCs/CP-T cells) recipients following ragweed exposure and the frequency of cells was determined by flow cytometry. Each experiment included 6–8 animals per group and experiment replicated at least three times. Error bars indicated the standard error of mean. IP group compared with ragweed group, ***p < 0.001 were considered significant. [file 13223_2021_542_MOESM1_ESM.pptx]

## Slide 1
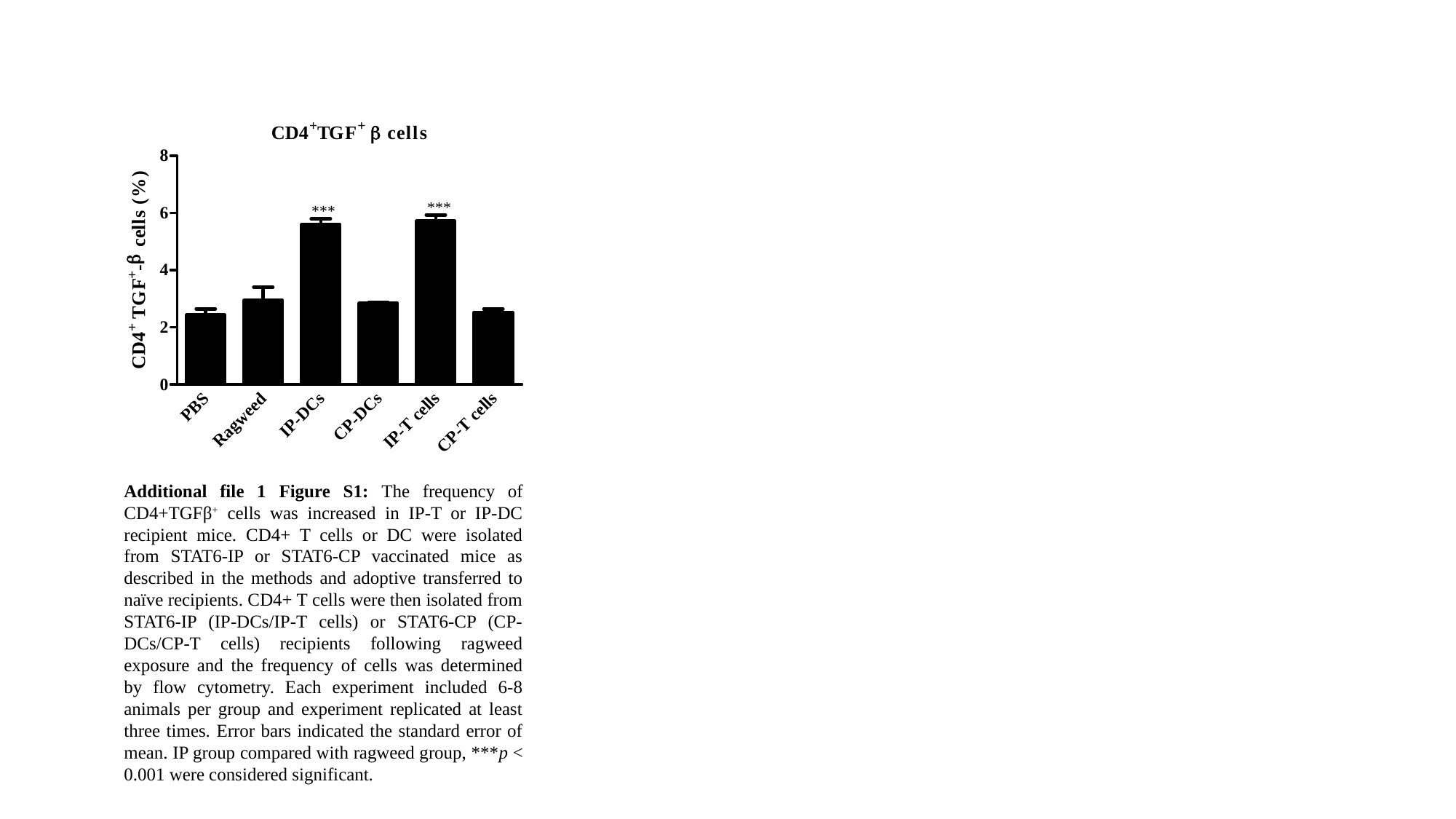

Additional file 1 Figure S1: The frequency of CD4+TGFβ+ cells was increased in IP-T or IP-DC recipient mice. CD4+ T cells or DC were isolated from STAT6-IP or STAT6-CP vaccinated mice as described in the methods and adoptive transferred to naïve recipients. CD4+ T cells were then isolated from STAT6-IP (IP-DCs/IP-T cells) or STAT6-CP (CP-DCs/CP-T cells) recipients following ragweed exposure and the frequency of cells was determined by flow cytometry. Each experiment included 6-8 animals per group and experiment replicated at least three times. Error bars indicated the standard error of mean. IP group compared with ragweed group, ***p < 0.001 were considered significant.
